# Blocking TLR7- and TLR9-mediated IFN-α Production by Plasmacytoid Dendritic Cells Does Not Diminish Immune Activation in Early SIV Infection

**DOI:** 10.1371/journal.ppat.1003530

**Published:** 2013-07-25

**Authors:** Muhamuda Kader, Amanda P. Smith, Cristiana Guiducci, Elizabeth R. Wonderlich, Daniel Normolle, Simon C. Watkins, Franck J. Barrat, Simon M. Barratt-Boyes

**Affiliations:** 1 Center for Vaccine Research, University of Pittsburgh, Pittsburgh, Pennsylvania, United States of America; 2 Department of Infectious Diseases and Microbiology, University of Pittsburgh, Pittsburgh, Pennsylvania, United States of America; 3 Dynavax Technologies Corporation, Berkeley, California, United States of America; 4 Department of Biostatistics, University of Pittsburgh, Pittsburgh, Pennsylvania, United States of America; 5 Department of Cell Biology and Physiology, University of Pittsburgh, Pittsburgh, Pennsylvania, United States of America; 6 Department of Immunology, University of Pittsburgh, Pittsburgh, Pennsylvania, United States of America; Emory University, United States of America

## Abstract

Persistent production of type I interferon (IFN) by activated plasmacytoid dendritic cells (pDC) is a leading model to explain chronic immune activation in human immunodeficiency virus (HIV) infection but direct evidence for this is lacking. We used a dual antagonist of Toll-like receptor (TLR) 7 and TLR9 to selectively inhibit responses of pDC but not other mononuclear phagocytes to viral RNA prior to and for 8 weeks following pathogenic simian immunodeficiency virus (SIV) infection of rhesus macaques. We show that pDC are major but not exclusive producers of IFN-α that rapidly become unresponsive to virus stimulation following SIV infection, whereas myeloid DC gain the capacity to produce IFN-α, albeit at low levels. pDC mediate a marked but transient IFN-α response in lymph nodes during the acute phase that is blocked by administration of TLR7 and TLR9 antagonist without impacting pDC recruitment. TLR7 and TLR9 blockade did not impact virus load or the acute IFN-α response in plasma and had minimal effect on expression of IFN-stimulated genes in both blood and lymph node. TLR7 and TLR9 blockade did not prevent activation of memory CD4+ and CD8+ T cells in blood or lymph node but led to significant increases in proliferation of both subsets in blood following SIV infection. Our findings reveal that virus-mediated activation of pDC through TLR7 and TLR9 contributes to substantial but transient IFN-α production following pathogenic SIV infection. However, the data indicate that pDC activation and IFN-α production are unlikely to be major factors in driving immune activation in early infection. Based on these findings therapeutic strategies aimed at blocking pDC function and IFN-α production may not reduce HIV-associated immunopathology.

## Author Summary

A persistent type I interferon (IFN) response is thought to be important in driving immune activation and progression to AIDS in human immunodeficiency virus (HIV)-infected individuals. Plasmacytoid dendritic cells (pDC) produce copious amounts of type I IFN upon virus exposure through engagement of Toll-like receptor (TLR) 7 and TLR9 and thus may be central players in the etiology of immune activation. We used a dual antagonist of TLR7 and TLR9 to selectively block the response of pDC but not other mononuclear phagocytes prior to and for 8 weeks following simian immunodeficiency virus (SIV) infection of rhesus macaques. We show that pDC are major, but not exclusive, producers of IFN-α that mediate a marked but transient IFN-α response in lymph nodes in the acute phase of infection. TLR7 and TLR9 antagonist prevented this IFN-α production without suppressing pDC recruitment. Nevertheless, TLR7 and TLR9 blockade did not impact expression of IFN-stimulated genes or decrease the activation of T cells, the hallmarks of immune activation. The findings indicate that TLR7 and TLR9-driven activation of pDC is unlikely to be a major contributor to immune activation in the early stages of immunodeficiency virus infections and suggest that therapeutic strategies aimed at targeting pDC and IFN-α production may not reduce HIV-associated immunopathology.

## Introduction

Chronic immune activation is a driving factor in CD4+ T cell loss and disease progression in HIV-infected individuals, yet the mechanisms responsible for this process are not completely understood [1]. Recent comparative studies in nonhuman primate models have shed light on the etiology of chronic immune activation [2]. Pathogenic simian immunodeficiency virus (SIV) infection in non-natural hosts including the Asian macaque species is characterized by sustained depletion of peripheral and mucosal CD4+ T cells, microbial translocation across the gut mucosa and persistently high levels of proinflammatory cytokines and lymphocyte activation that culminate in disease progression and AIDS [3]–[7]. In contrast, SIV infection of natural hosts such as the African green monkey and sooty mangabey results in preserved T cell homeostasis, low levels of chronic immune activation and a benign clinical course despite high levels of circulating virus [8]–[11]. A key distinction between the two models is that the innate immune response is rapidly resolved in SIV-infected natural hosts, whereas upregulation of the type I interferon (IFN) response and expression of IFN-stimulated genes (ISG) persists in SIV-infected macaques [12]–[17]. This dichotomy suggests that the innate immune response and persistent type I IFN production in particular may play a key role in chronic immune activation and disease progression [18], [19].

Plasmacytoid dendritic cells (pDC) produce copious amounts of type I IFN in response to virus exposure but their role in HIV infection appears to be complex [20]. pDC are activated in HIV and SIV infection and are rapidly lost from blood, coincident with their recruitment to lymph nodes and mucosal tissues [21]–[27], and within acutely infected lymph nodes IFN-α is produced largely by pDC [16], [17]. In addition, pDC may be chronically stimulated in HIV infection and be a continuing source of IFN-α that leads to CD4 T cell death [28]–[32]. These findings have led to a model in which activated pDC that are recruited to lymphoid tissues chronically produce IFN-α that drives sustained expression of ISG and mediates T cell dysfunction and loss [18], [33]. However, to date a direct link between the pDC response and chronic immune activation has not been made as reagents that selectively deplete pDC or interfere with their function in nonhuman primates have not been available. Resolving this issue has important clinical implications as therapeutic strategies aimed at disrupting pDC function are being considered as a means of controlling HIV-associated immunopathology [34], [35].

pDC are activated by HIV and SIV nucleic acids to produce IFN-α and TNF-α through engagement of endosomal receptors TLR7 and TLR9 [36], [37], and dual antagonists comprised of nonstimulatory DNA sequences have been developed that block pDC stimulation through these receptors [38]. These compounds have been used to demonstrate the critical contribution of pDC to systemic lupus erythematosis and skin autoimmune disease in mouse models [39], [40]. In the present study, we used a dual TLR7 and TLR9 antagonist to selectively block the response of pDC but not other mononuclear phagocytes to viral RNA in SIV-infected rhesus macaques and directly determine the role of pDC in immune activation.

## Results

### TLR7 and TLR9 antagonist has selective activity against rhesus macaque pDC

**Figure 1:**
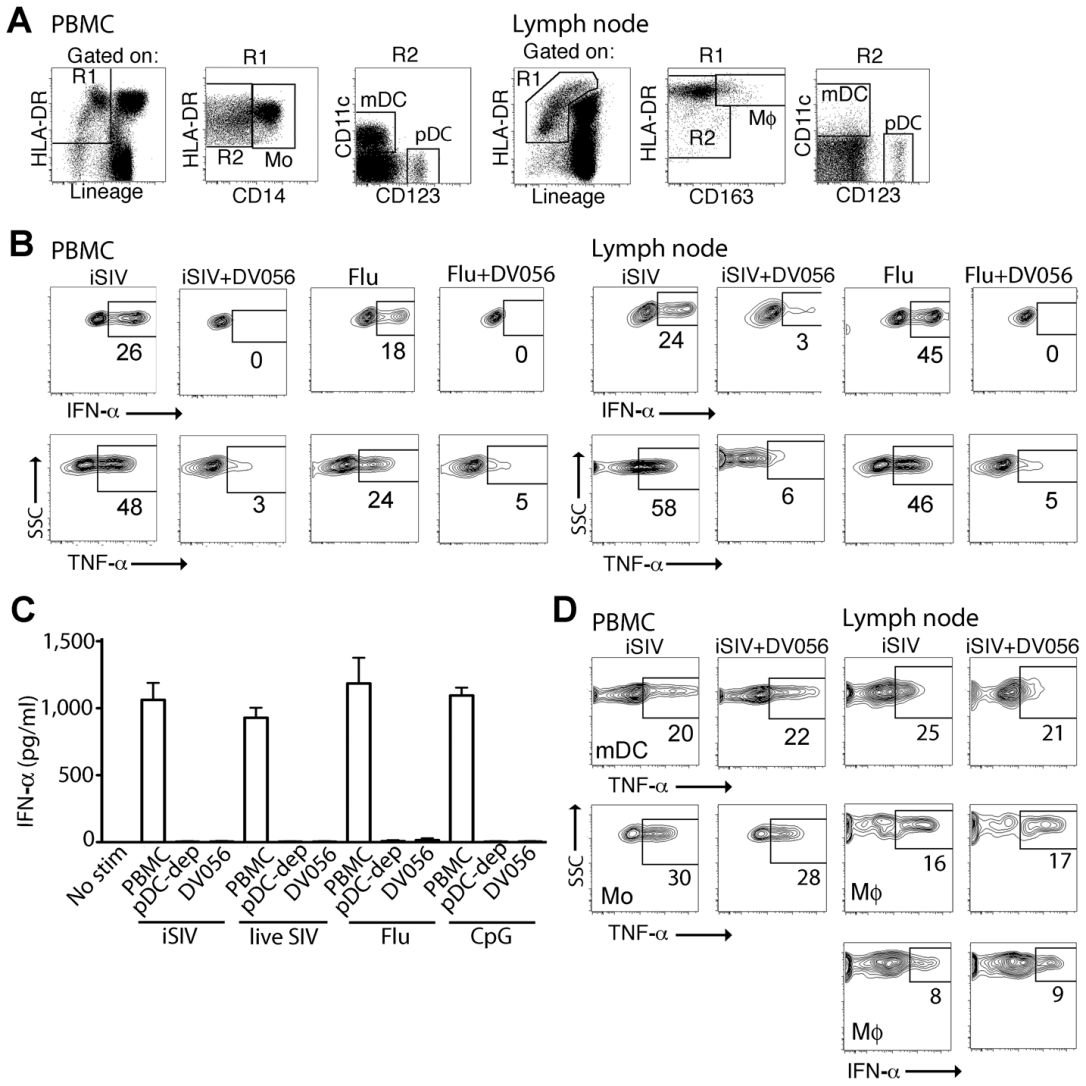
TLR7 and TLR9 antagonist selectively blocks proinflammatory cytokine production from uninfected rhesus macaque pDC. (A) Representative gating strategy to define pDC, mDC, monocytes (Mo) and macrophages (Mø) by flow cytometry in rhesus macaque PBMC and lymph node. The lymph node gate to define DC includes autofluorescent cells that encroach on the Lineage+ subset, as previously described [23]. (B) PBMC and lymph node cell suspensions from SIV-naive animals were stimulated with iSIV or influenza virus with and without DV056 and intracellular expression of IFN-α and TNF-α in pDC was determined by flow cytometry. Positive labeling with cytokine-specific antibody as shown by the boxed areas was based on isotype control antibody. Numbers represent the percent positive cells. (C) PBMC, pDC-depleted PBMC and PBMC pretreated with DV056 were stimulated with iSIV, live SIV, influenza virus or CpG-C and supernatants were analyzed for IFN-α by ELISA. Unstimulated PBMC served as a negative control. (D) PBMC and lymph node cell suspensions were stimulated with iSIV or influenza virus and IFN-α and/or TNF-α in mDC, monocytes or macrophages were determined by flow cytometry. SSC = side scatter.

To determine if TLR7 and TLR9 blockade was effective at selectively inhibiting pDC responses to SIV in rhesus macaques, we first tested the specificity and efficacy of a TLR7 and TLR9 antagonist in vitro. Peripheral blood mononuclear cells (PBMC) and peripheral lymph node cell suspensions from healthy SIV-naïve macaques were incubated with aldrithiol-2-inactivated SIVmac239 particles (iSIV) or influenza virus in the presence or absence of DV056, a 25-based single-stranded phosphorothioate oligodeoxynucleotide antagonist of TLR7 and TLR9 that contains the necessary inhibitory motifs with optimization for activity in nonhuman primates and humans [37]. After incubation cytokine production was determined by flow cytometry using standard approaches to define mononuclear phagocytes (Figure 1A) [23], [41]. Blood and lymph node pDC produced abundant IFN-α and TNF-α in response to iSIV and influenza virus, which was potently blocked by DV056 (Figure 1B). pDC were the exclusive producers of IFN-α in SIV-naive blood in response to stimulation with iSIV, live SIV, influenza virus and the TLR9 agonist CpG-C, as shown by the lack of IFN-α secretion from stimulated PBMC that were depleted of pDC (Figure 1C). Moreover, DV056 completely blocked secretion of IFN-α from unseparated PBMC in response to these varied stimuli, demonstrating the activity of the drug in antagonizing both TLR7 and TLR9 (Figure 1C). In contrast, DV056 did not impair TNF-α production from blood and lymph node myeloid DC (mDC) and blood monocytes or TNF-α and IFN-α production from lymph node macrophages in response to stimulation with iSIV, consistent with the fact that these cells recognize viral RNA through receptors other than TLR7 and TLR9 (Figure 1D) [42].

### In vivo delivery of TLR7 and TLR9 antagonist blocks responsiveness of rhesus blood and lymph node pDC to virus stimulation

**Figure 2:**
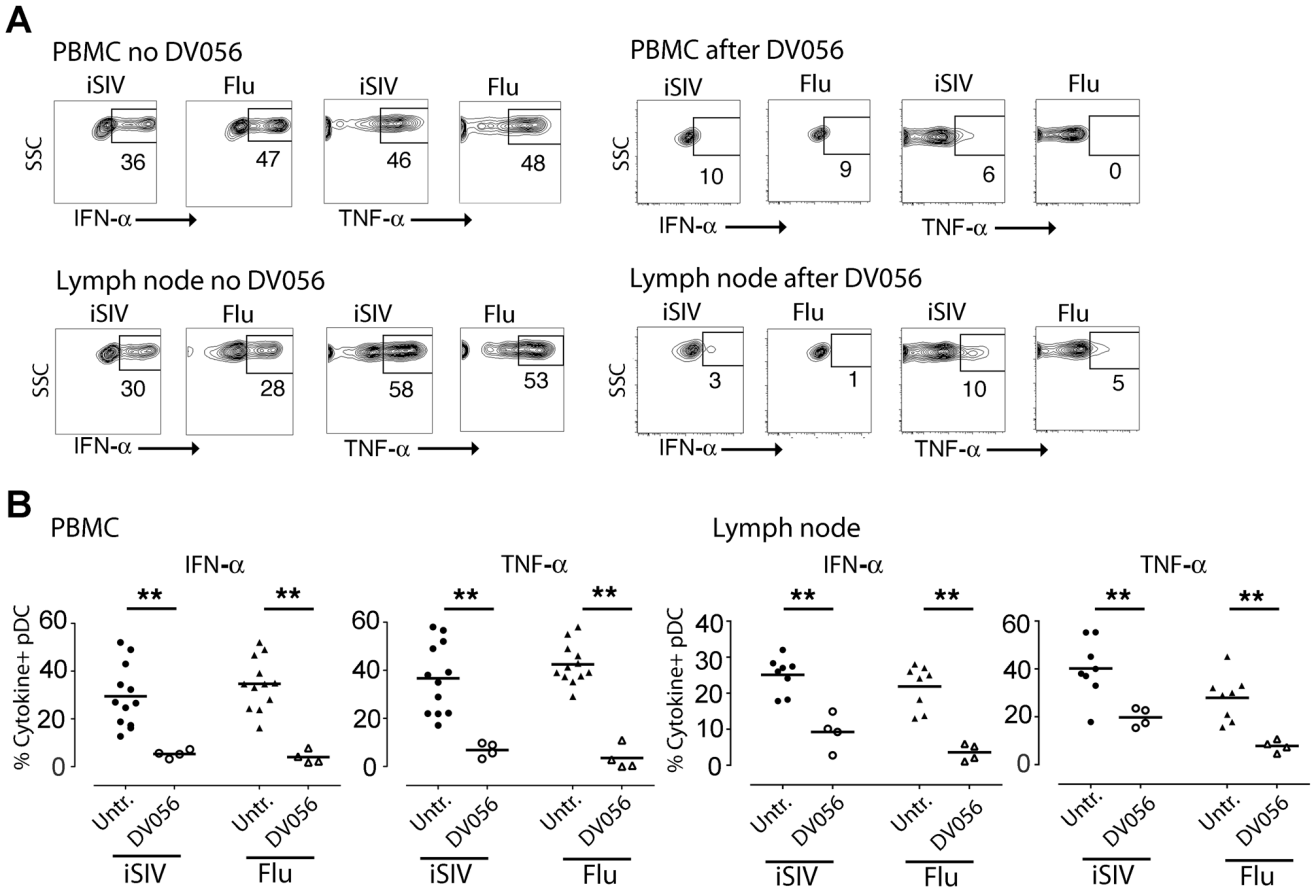
In vivo administration of TLR7 and TLR9 antagonist to rhesus macaques blocks pDC responses to virus stimulation. DV056 was administered by weekly subcutaneous injection at 2 mg/kg to SIV-naive rhesus macaques, and PBMC and lymph node cell suspensions collected 3 days after administration were stimulated with iSIV and influenza virus to assess TLR7 and TLR9 blockade. (A) pDC production of IFN-α and TNF-α in PBMC and lymph node cell suspensions from untreated and DV056-treated macaques. Flow cytometric gating was done as in Figure 1. (B) The percent of pDC in PBMC (left) and lymph node cell suspensions (right) from DV056-treated macaques (n = 4) compared to untreated macaques (n = 12 for PBMC, n = 8 for lymph node) producing IFN-α and TNF-α in response to iSIV and influenza virus stimulation. Each symbol represents an individual animal and horizontal lines represent means. **P<.01.

We next determined the capacity of DV056 to block the response to viral RNA in rhesus macaques in vivo. We treated 4 SIV-naive macaques with DV056 at a dose of 2 mg/kg by weekly subcutaneous injections and collected PBMC 3 days after each injection for functional analyses. This drug regimen was based on preliminary studies showing that a similar dose and course of a related TLR7 and TLR9 antagonist in macaques blocked the ex vivo induction of ISG in PBMC in response to influenza virus stimulation (F.J.B., unpublished data). After a single treatment the proportion of blood pDC producing cytokines in response to virus stimulation dropped by 80 to 90%. In addition, the amount of each cytokine produced by pDC as judged by mean fluorescence intensity was substantially reduced in DV056-treated macaques (Figure 2A, B). To assess the level of blockade in lymphoid tissues we harvested inguinal lymph nodes 3 days after the third dose of DV056 to allow time for accumulation of drug in tissues. The mean proportion of lymph node pDC producing IFN-α in DV056 treated animals dropped by 66% and 85% following stimulation with iSIV and influenza virus, respectively, relative to untreated lymph nodes, accompanied by marked reductions in the amount of cytokine produced. Reductions in lymph node pDC production of TNF-α ranged from 54% to 75% for iSIV and influenza virus, respectively (Figure 2A, B). The responsiveness of mDC and monocytes/macrophages in each compartment was unaffected by DV056 treatment (data not shown).

### TLR7 and TLR9 blockade does not alter virus load or pDC kinetics following SIV infection

**Figure 3:**
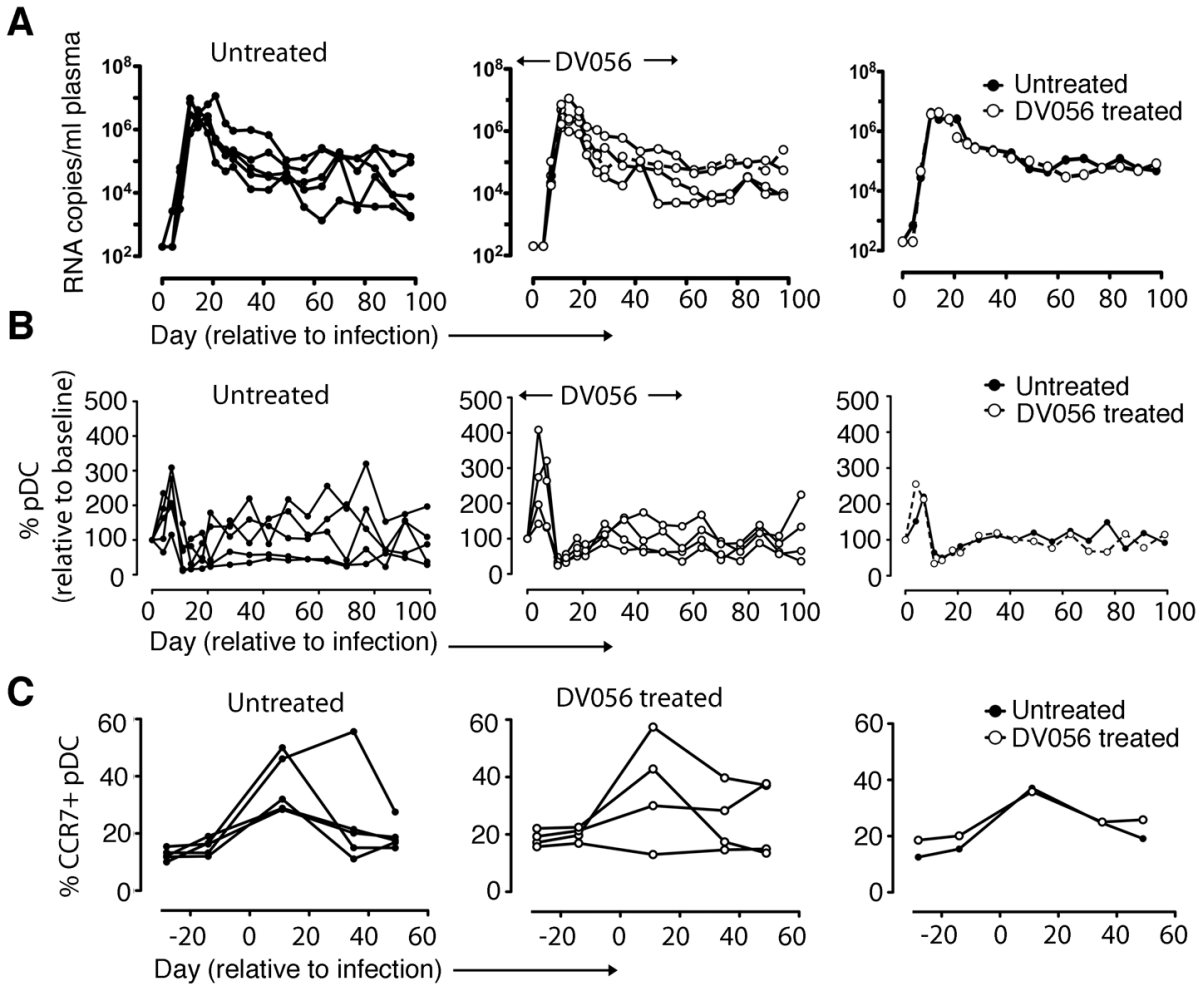
TLR7 and TLR9 blockade does not impact virus load or pDC kinetics. (A) SIV RNA copies/ml of plasma for untreated (n = 5) and DV056-treated (n = 4) macaques over time as determined by quantitative RT-PCR. Shown are virus loads for each animal in untreated and DV056-treated groups as well as geometric means for each group. (B) Absolute counts of pDC in blood for untreated and DV056-treated macaques over time. Shown is the percent change in cell counts relative to preinfection levels for each animal in untreated and DV056-treated groups as well as means for each group. (C) Percent of CCR7+ pDC in blood over time. Shown is the percent pDC expressing CCR7 before and after SIV infection for each animal in untreated and DV056-treated groups as well as the means for each group.

After a total of 4 weekly doses of DV056 we infected the 4 rhesus macaques with SIVmac251 intravenously and continued weekly drug dosing up to day 53 post infection, encompassing the critical acute-to-chronic phase when immune activation is established. In parallel we infected 5 untreated macaques with the same dose of virus as controls. The kinetics of viremia were highly similar in both groups of animals, with nearly identical peak virus loads at day 11 and virus set-points at around day 70 post infection (Figure 3A). Infection was associated with a marked increase and then decline in the number of blood pDC which then steadily returned to pre-infection levels by around day 30 post infection, and these kinetics were unaffected by DV056 treatment (Figure 3B). Acute infection was also associated with a significant increase in the proportion of pDC expressing the chemokine receptor CCR7 regardless of DV056 treatment, consistent with the potential for blood pDC to migrate to lymph nodes (Figure 3C).

### TLR7 and TLR9 antagonist blocks pDC responses in SIV-infected macaques

**Figure 4:**
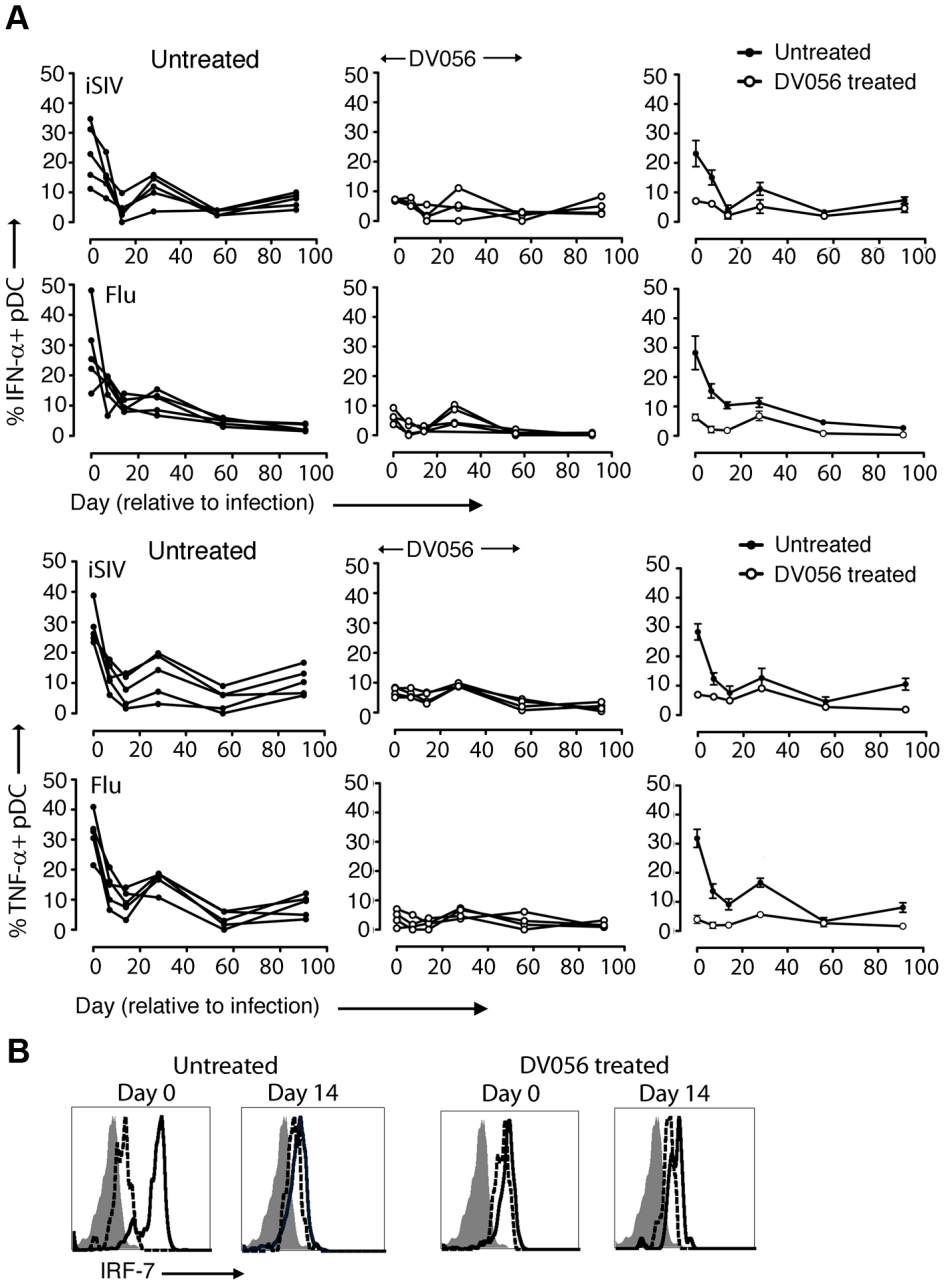
TLR7 and TLR9 antagonist blocks pDC responses following SIV infection. (A) Percent of blood pDC producing IFN-α and TNF-α after stimulation with iSIV and influenza virus following SIVmac251 infection. Shown is the percent of cytokine-expressing pDC for each animal in untreated (n = 5) and DV056-treated (n = 4) groups as well as means for each group. The difference between untreated and DV056-treated profiles was statistically significant for both viruses and both cytokines (*P*<.01). (B) IRF-7 expression in pDC from an untreated and DV056-treated macaque at day 0 and day 14 post infection after stimulation with iSIV (solid line) or no stimulation (dotted line) relative to isotype control antibody (filled histogram).

**Figure 5:**
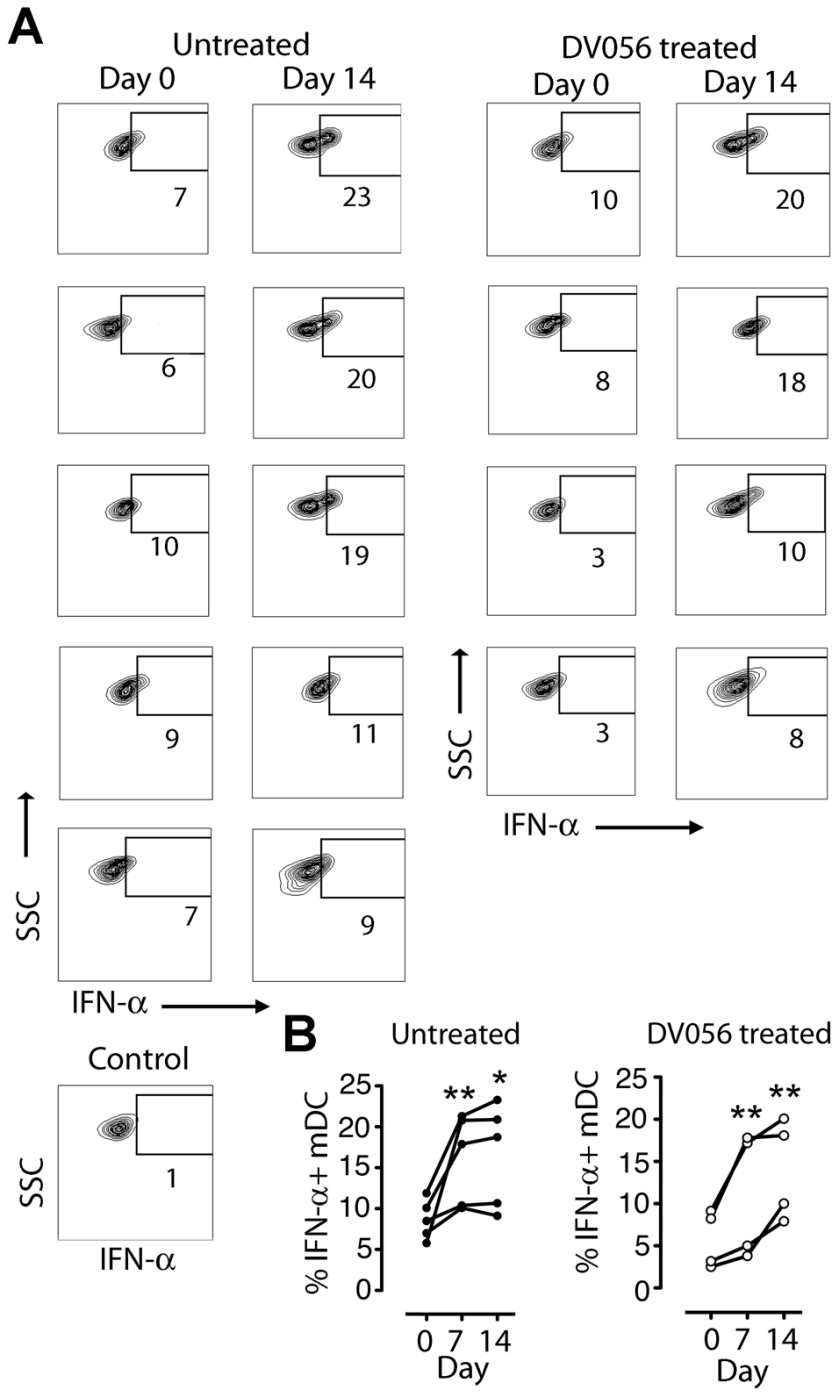
mDC gain the capacity to secrete IFN-α upon virus stimulation following SIV infection. (A) Dot plots showing intracellular IFN-α expression in blood mDC from untreated and DV056-treated macaques at day 0 and day 14 post infection after iSIV stimulation. Data for all animals are shown, along with a representative dot plot of cells exposed to control microvesicles. Flow cytometric gating to define mDC was done as in Figure 1. (B) Percent of blood mDC producing IFN-α after iSIV stimulation at the indicated times after infection in untreated and DV056-treated macaques. *P<.05, **P<.01.

We next harvested PBMC from DV056-treated and control macaques and measured proinflammatory cytokine production after virus exposure ex vivo. Blood pDC from macaques that did not receive DV056 treatment had robust IFN-α and TNF-α production when stimulated with iSIV and influenza virus at the time of SIV infection. However, pDC from these animals rapidly lost responsiveness to virus stimulation ex vivo, and pDC hyporesponsiveness persisted into chronic infection, consistent with previous studies (Figure 4A) [43]. pDC from monkeys in the DV056-treated group had suppressed responses to virus stimulation at the time of SIVmac251 infection, revealing that TLR7 and TLR9 blockade remained effective even after multiple drug administrations, and this suppression was sustained after infection and persisted for the length of the study (Figure 4A). In addition, expression of IRF-7, a key IFN-α transcription factor that is induced in pDC upon HIV stimulation [32], [44], was markedly upregulated in iSIV-stimulated blood pDC from untreated macaques at day 0 but not at day 14 post infection, and was not upregulated in pDC from DV056-treated macaques at either time, consistent with both virus- and drug-induced inhibition of pDC responses (Figure 4B). In contrast to pDC, blood mDC gained significant capacity to produce IFN-α in acute SIV infection when stimulated with iSIV ex vivo, although the intensity of cytokine production based on mean fluorescence intensity was relatively modest and there was considerable variation between animals (Figure 5A, B). Notably, the enhancement of mDC function was not blocked by DV056 treatment, consistent with the inability of TLR7 and TLR9 antagonist to impact mDC responses to viral RNA (Figure 5A, B).

### IFN-α and TNF-α are differentially expressed and impacted by TLR7 and TLR9 blockade in lymph nodes

**Figure 6:**
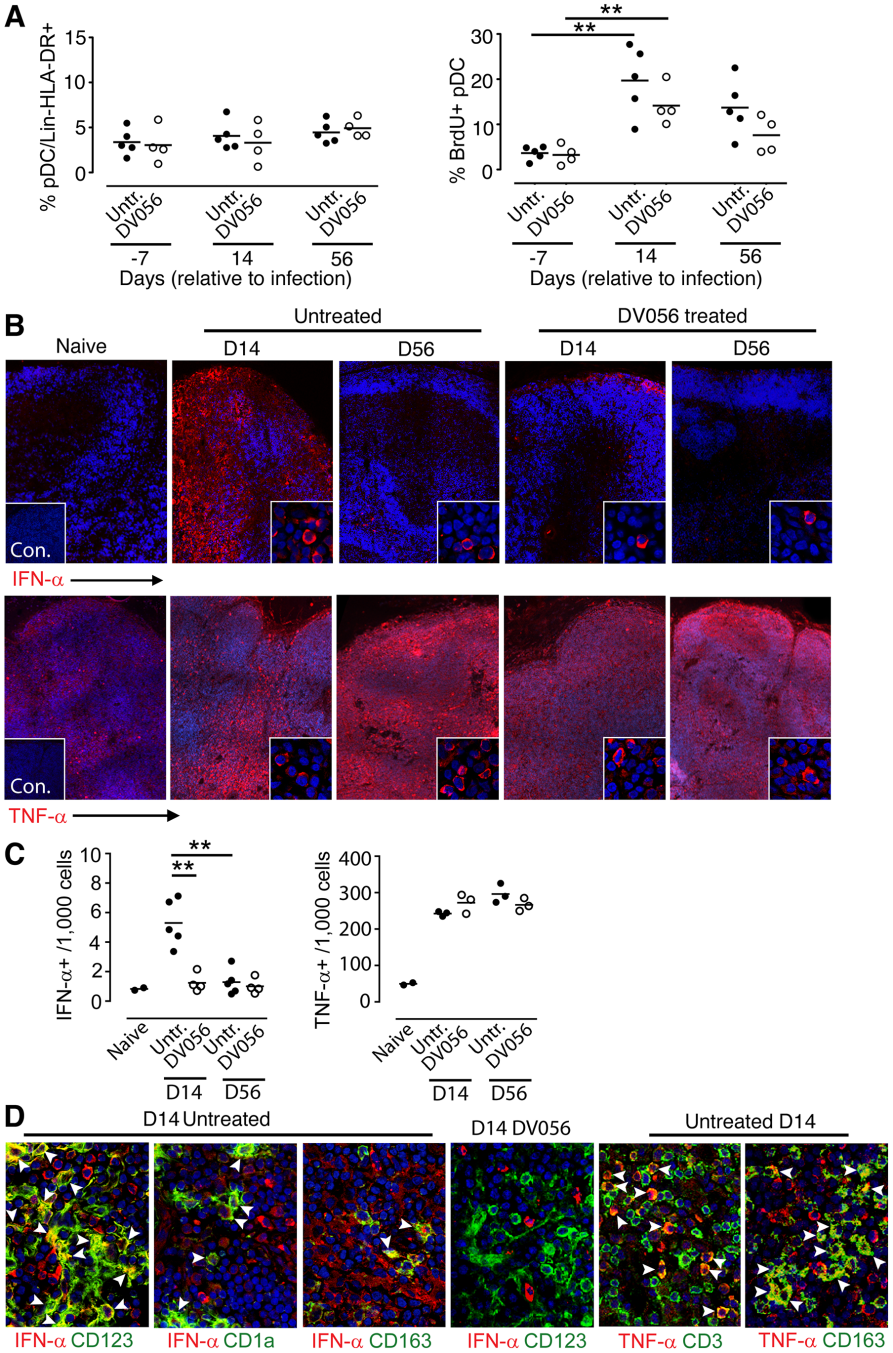
TLR7 and TLR9 antagonist blocks transient IFN-α production by pDC in lymph nodes without suppressing recruitment. (A) Percent of pDC in lymph nodes (left) and percent of pDC labeling with antibody to BrdU (right) in untreated (n = 5) and DV056-treated (n = 4) macaques. Horizontal lines represent means. **P<.01. (B) Immunofluorescence of lymph node sections taken from naive and SIV-infected macaques with and without DV056 treatment at days 14 and 56 post infection stained with antibody to IFN-α or TNF-α (red) and co-stained with Hoescht dye (blue) to identify nuclei. Insets in naïve tissue sections represent immunofluorescence after labeling with isotype control antibody. Insets in SIV-infected sections show higher magnification images to highlight details of individual cells. Original magnification 400×. (C) Frequency of IFN-α and TNF-α producing cells in paracortex and parafollicular cortex from naive macaques (n = 2) and macaques at day 14 and 56 post infection with (n = 4 for IFN-α, n = 3 for TNF-α) and without (n = 5 for IFN-α, n = 3 for TNF-α) DV056 treatment. **P<.01. (D) Immunofluorescence of lymph nodes from untreated and DV056-treated macaques at day 14 post infection stained with antibody against CD123, CD1a or CD163 (green) and IFN-α or TNF-α (red) and co-stained with Hoescht dye (blue). Arrowheads indicate cells co-labeling with cytokine- and cell-specific markers. Original magnification 400×.

We next determined if DV056 treatment impacted pDC recruitment and cytokine production in lymph nodes following SIV infection. The frequency of CD123+ pDC within the Lineage– HLA-DR+ fraction of cells did not change at day 14 or 56 post infection in either DV056-treated or control animals when compared to preinfection samples (Fig. 6A). However, the proportion of lymph node pDC that was BrdU+ increased significantly at day 14 post infection in both groups, reflecting the influx of recently divided pDC to lymph nodes [25] that occurred regardless of TLR7 and TLR9 blockade (Fig. 6A). We next analyzed serial sections of lymph nodes for in situ expression of IFN-α and TNF-α, quantifying the frequency of cytokine-expressing cells using imaging software (Figure S1). In lymph nodes from untreated macaques at day 14 post infection IFN-α-expressing cells were frequent in lymph nodes, with an average of 0.5% of all cells in the paracortex and parafollicular cortex producing IFN-α. This expression was transient, as by day 56 post infection IFN-α-producing cells were rarely identified and their frequencies approximated those of naive lymph nodes (Fig. 6B,C). In contrast, animals receiving DV056 treatment did not experience this transient increase in IFN-α production in acute infection; IFN-α-producing cells were rare in the paracortex and parafollicular cortex at both day 14 and 56 post infection with frequencies similar to that seen in naive macaques (Fig. 6B,C). A majority but not all of the IFN-α expressing cells in untreated lymph nodes co-expressed CD123, indicating that pDC were major but not exclusive producers of IFN-α in the absence of DV056 treatment. Additional double labeling experiments revealed IFN-α production by CD163+ macrophages and by mDC, identified using antibody to CD1a (Fig. 6D) [23]. IFN-α in lymph nodes from DV056-treated macaques did not co-stain with antibody to CD123 but was largely restricted to macrophages and mDC (Fig. 6D and data not shown). In contrast to IFN-α, massive numbers of TNF-α-producing cells were present in lymph nodes at both day 14 and 56 post infection, regardless of TLR7 and TLR9 blockade, with 25 to 30% of all cells in the paracortex and parafollicular cortex expressing this cytokine (Fig. 6B,C). TNF-α producing cells were primarily CD163+ macrophages and CD3+ T cells (Fig. 6D). CD123+ pDC that co-labeled with antibody to TNF-α were rarely identified, even in the untreated animals (data not shown).

### TLR7 and TLR9 blockade has minimal impact on plasma IFN-α and expression of ISG after SIV infection

**Figure 7:**
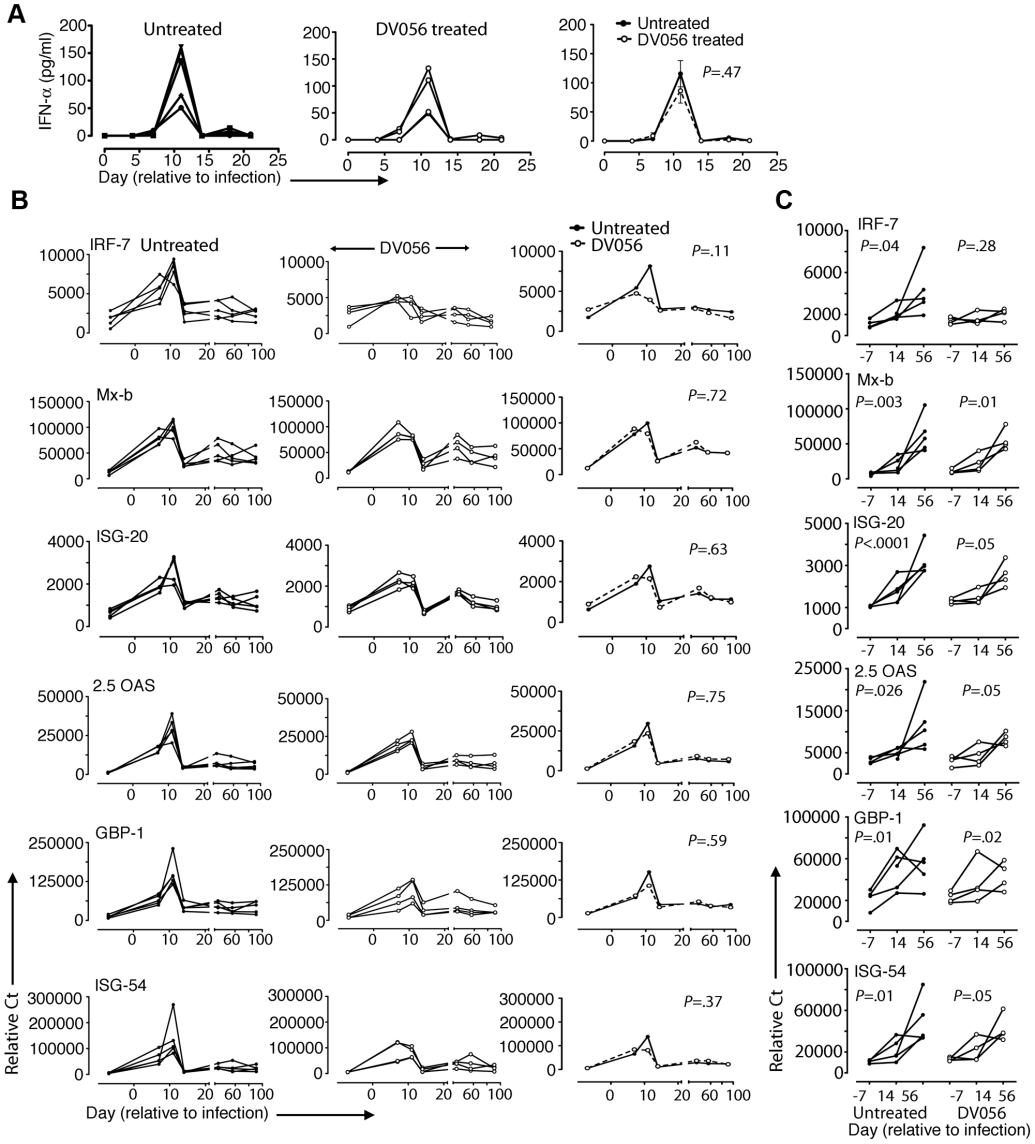
Minimal impact of TLR7 and TLR9 blockade on plasma IFN-α and ISG expression. (A) Plasma IFN-α levels were quantified by ELISA. Shown is the plasma concentration of IFN-α for each animal in untreated (n = 5) and DV056-treated (n = 4) groups as well as the means for each group. (B) Relative expression of ISG in PBMC. Shown is the expression of the indicated genes normalized to ubiquitin for each animal in untreated and DV056-treated groups as well as the means for each group over time. *P* values in A and B reflect differences between untreated and DV056 treated profiles. (C) Expression of the same genes in lymph nodes. Shown is the mean +/− SEM for individual genes in lymph node cell suspensions for untreated (n = 5) and DV056-treated (n = 4) macaques. *P* values reflect trends in gene expression over the 3 time points.

We next evaluated the impact that DV056 treatment had on the systemic type I IFN response following infection. In macaques that were SIV-infected without concurrent TLR7 and TLR9 blockade we detected a robust and transient increase in plasma levels of IFN-α which peaked at day 11 post infection. DV056-treated macaques had nearly identical plasma IFN-α responses (Figure 7A). To determine the impact that TLR7 and TLR9 blockade had on ISG expression, we analyzed the relative expression of 6 genes (IRF-7, Mx-b, ISG-20, 2.5 OAS, GBP-1 and ISG-54) that are induced in blood and lymph node CD4+ T cells in SIV-infected macaques [14], [15]. In PBMC from untreated animals ISG responses paralleled those of plasma IFN-α, peaking at day 11 post infection and then dropping to near pre-infection levels (Figure 7B). Induction of expression of all ISG examined was impacted minimally or not at all by DV056 treatment (Figure 7B). In lymph node cell suspensions of untreated macaques, expression of ISG increased progressively from day 14 to day 56, in contrast to blood (Figure 7C). Treatment with DV056 blocked the induction of IRF-7 but otherwise did not diminish expression of ISG in lymph nodes subsequent to SIV infection (Figure 7C).

### TLR7 and TLR9 blockade does not diminish CD4+ and CD8+ memory T cell activation and proliferation in blood or lymph node

**Figure 8:**
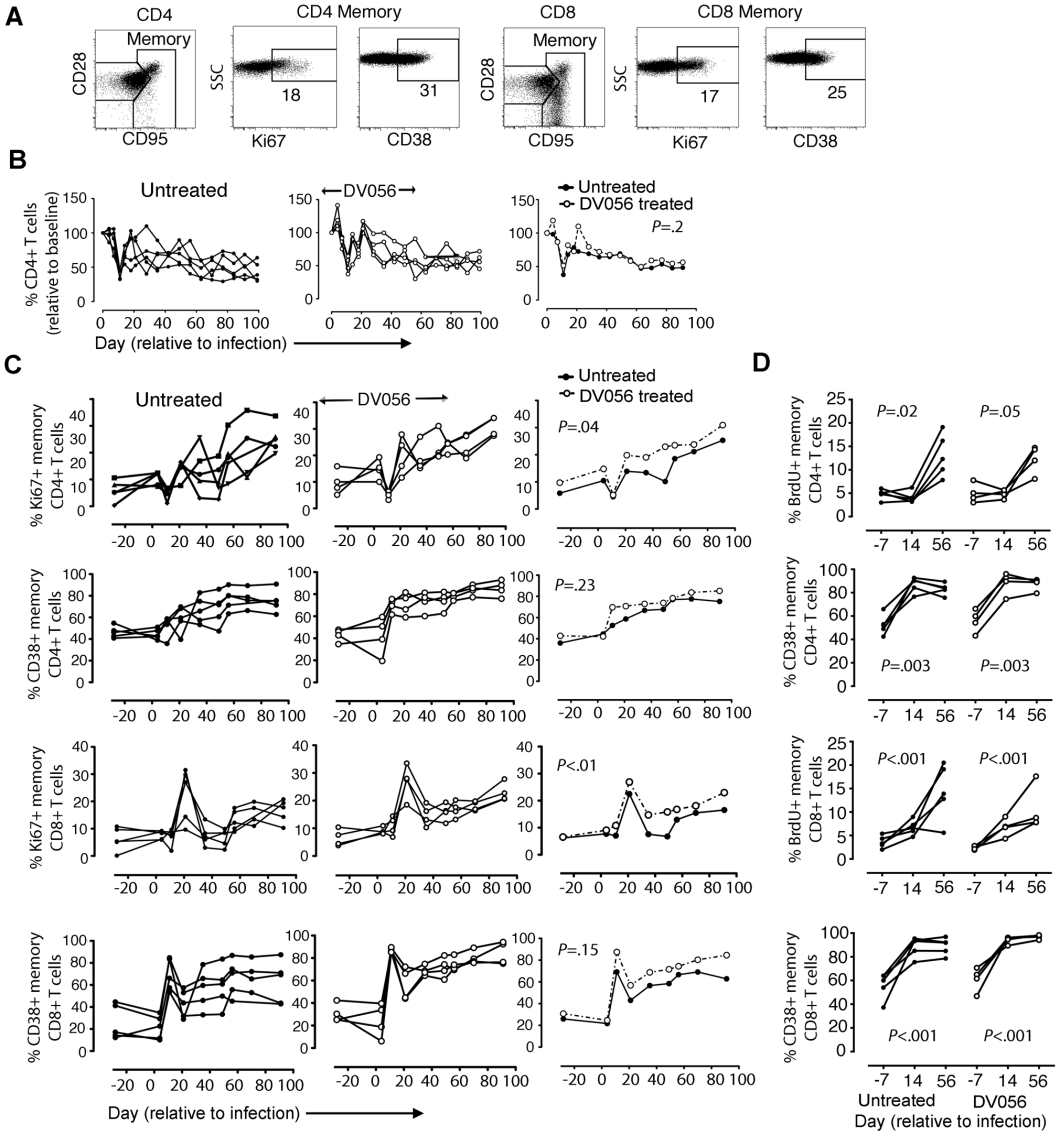
TLR7 and TLR9 blockade does not diminish immune activation in SIV-infected rhesus macaques. (A) Representative gating strategy to define CD4+ and CD8+ T cell subsets in rhesus macaque PBMC. (B) Absolute counts of CD4+ T cells in blood of untreated (n = 5) and DV056-treated (n = 4) macaques over time. Shown is the percent change in cell counts relative to preinfection levels for each animal in untreated and treated groups as well as the means for each group. (C) Percent of CD4+ and CD8+ memory T cells in blood expressing Ki67 and CD38. Shown is the percent expression for each animal in untreated and treated groups as well as means for each group. *P* values in B and C reflect differences between untreated and DV056 treated profiles. (D) Percent of CD4+ and CD8+memory T cells in lymph nodes expressing Ki67 and CD38. Shown are mean +/− SEM for all animals in each group. *P* values reflect trends in the frequency of cells over the 3 time points.

To determine if T cell activation and proliferation were impacted by TLR7 and TLR9 blockade, we analyzed CD4+ and CD8+ T cell subsets in blood and lymph node cell suspensions in DV056-treated and untreated macaques by flow cytometry (Figure 8A). We focused on the memory subsets characterized by high expression of CD95 with and without expression of CD28, as dysregulation is most prominent in these cells [45]. The number of CD4+ T cells in peripheral blood rapidly and transiently declined at day 11 post infection before rebounding and then steadily declining in untreated macaques. In macaques treated with DV056 blood CD4 T cell counts were transiently increased at day 4 and day 21 post infection, although the pattern of changes in CD4+ T cell counts over time was not significantly different between groups (Figure 8B). In untreated macaques SIV infection resulted in the continual increase in frequency of memory CD4+ and memory CD8+ T cells in blood expressing Ki67, an indicator of recent proliferation, and the activation marker CD38 [46]. The frequency of memory T cells expressing CD38 was not altered in DV056 treated macaques, while the frequency of both memory T cell subsets expressing Ki67 actually increased over time as a result of DV056 treatment (Figure 8C). In inguinal lymph node cell suspensions the increase in proliferating memory T cell subsets following SIV infection, as determined by BrdU incorporation, was almost identical in control and DV056-treated macaques (Figure 8D). The frequency of memory T cell subsets in lymph nodes expressing the activation marker CD38 approached 90% to 100% in both groups indicating profound activation regardless of DV056 treatment (Figure 8D).

## Discussion

This study is the first to directly dissect the role of innate immunity in driving immune activation in pathogenic SIV infection of rhesus macaques, a model that produces AIDS-like disease very similar to HIV infection in humans but with an accelerated time frame [47]. We have demonstrated that stimulation of pDC by viral RNA through engagement of TLR7 and TLR9 induces a robust but transient IFN-α response in the lymph nodes of SIV-infected rhesus macaques, and provide evidence that this response in itself is insufficient to drive persistent ISG expression and immune activation that distinguishes pathogenic from nonpathogenic models [13]–[15].

While pDC were found to be major producers of IFN-α in response to SIV infection, they were not exclusive producers of this cytokine. Macrophages from naïve macaques produced IFN-α when stimulated in vitro with iSIV and were found to contribute to IFN-α production in lymph nodes during the acute phase of infection, although as shown here for pDC, macrophages and monocytes also become refractory to stimulation following SIV infection [43]. Conversely, while mDC from SIV naïve animals are largely incapable of producing IFN-α upon stimulation with SIV or virus-encoded oligonucleotides [43], we found that mDC taken after infection gained a modest capacity to produce IFN-α upon virus stimulation and also contributed to in situ-IFN-α production in acutely infected lymph nodes. Similarly, in humans with chronic HIV infection, macrophages, mDC and lymphocytes produce IFN-α in the spleen and appear to be greater overall contributors to IFN-α production than pDC [48]. These findings reflect the fact that multiple cell types including hematopoietic and non-hematopoietic cells produce type I IFN through both TLR-dependent and independent pathways, depending on the type of stimulus [49]. Indeed, in murine models of virus infection ablation of pDC impacts IFN-α production only in the very early stages after infection, up to 36 hours; non-pDC are responsible for IFN-α at later time points [50]. The redundancy in IFN-α production may explain why blocking TLR7 and TLR9 function did not substantially diminish the peak plasma IFN-α response or alter the kinetics of ISG expression in infected monkeys. In addition, it is possible that the dramatically reduced levels of IFN-α in lymph nodes of DV056-treated animals are nevertheless sufficient to induce the high levels of ISG observed in these tissues, or that other viral IFN including IFN-β and IFN-λ that were not measured in this study contribute to a robust ISG response in the face of TLR7 and TLR9 blockade [51].

Notably, IFN-α production in lymph nodes was transient despite sustained high levels of virus and was temporally unrelated to chronic immune activation. In other studies, deliberately increasing levels of IFN-α in chronically infected sooty mangabeys through exogenous injection of an IFN-α agonist does not induce lymphocyte activation but reduces virus load [52]. In addition, in individuals with chronic HIV infection systemic IFN-α administration reduces virus load even as it enhances CD8+ T cell activation [53], [54]. Collectively, these data do not support a direct pathologic role for IFN-α in disease progression in HIV infection. It is possible that chronic immune activation that is the hallmark of pathogenic SIV and HIV infection is more a consequence of sustained exposure to microbial products that have been translocated across the gut lumen, resulting in persistent activation of mononuclear phagocytes and other cells through the actions of lipopolysaccharide [7], [55]. TNF-α overproduction by a subset of blood monocytes in response to microbial products has been demonstrated in HIV viremic individuals, providing a mechanistic link between microbial translocation and systemic inflammation [56]. In our study copious in situ TNF-α production was temporally associated with ISG expression in lymph nodes, and other cytokines including TGF-β, IL-1β and IL-15 that are increased in lymph nodes in acute SIV and HIV infection may also play a role either directly or indirectly in immune activation and disease progression [57]–[59].

We were unable to sample gut mucosal tissues for pDC activity and TLR7 and TLR9 blockade and therefore cannot rule out the possibility that pDC recruited to these tissues maintained continuous production of IFN-α that drove ISG expression and immune activation [26], [27]. Phosphorothioate oligonucleotides have very predictable pharmacokinetics across species, with rapid clearance from plasma and accumulation primarily in lymphoid tissues, liver and kidney with a half-life of 2–6 days [60]–[63], and studies with a related TLR7 and TLR9 antagonist to DV056 indicate a similar profile (C.G. and F.J.B., unpublished data). These compounds also accumulate in intestine at levels 3–6 times that of blood [61], [64]. We would therefore predict that DV056 blocked pDC responses in mucosal tissues as it did in lymphoid tissues. Evidence also suggests that mucosal pDC become hyporesponsive to stimulation and IFN-α production subsequent to SIV infection [27], as they do in lymphoid tissues [43]. Further studies are needed to directly investigate the response of pDC and other mononuclear phagocytes in mucosal tissues and its relationship to disease progression.

TLR7 and TLR9 antagonist did not suppress the upregulation of CCR7 by pDC or their recruitment to lymph nodes in acutely infected macaques, despite the fact that HIV-mediated activation of TLR7 drives CCR7 upregulation in these cells [65]. This could be explained by the fact that the viral matrix protein alone induces functional CCR7 expression on pDC without inducing IFN-α [66], and this pathway would be intact in our study animals as TLR7 and TLR9 blockade did not reduce the amount of circulating virus. These findings reinforce those in the mouse model of autoimmune skin disease [40] and highlight the fact that pDC recruitment to inflamed tissues is not linked to their capacity to secrete proinflammatory cytokines.

An unexpected finding of our study was the significant increase in DV056-treated macaques of proliferating memory CD4+ and CD8+ T cells in blood. These increases were not associated with increased in virus load (data not shown) which is known to impact T cell proliferation [67], [68] and may therefore be a consequence of TLR7 and TLR9 blockade itself. The mechanism for this enhanced proliferative response and its biologic relevance is unclear at present. TLR-mediated activation of DC is known to impact regulatory T cell development and influence CD4+ T cell proliferation [69], [70], but the impact of selectively blocking TLR7 and TLR9 on T cell proliferation in the context of SIV or HIV infection has not been studied and deserves further attention.

Our findings support the conclusion that TLR7- and TLR9-mediated activation of pDC leads to significant and transient IFN-α production in lymph nodes but may not by itself drive the pathologic process of immune activation. The data reinforce the findings of others [16], [17] showing that production of IFN-α from activated pDC that have been recruited to lymph nodes in early infection does not define pathogenic SIV infection, contrary to earlier findings [37]. Our findings suggest that specifically targeting pDC as a therapeutic strategy to reduce immunopathology in HIV infection may have limited benefit [34], at least in the early stages of infection. It will be important to determine whether TLR7- and TLR9-driven activation of pDC plays any role in immune activation in the chronic stages of infection when disease is manifest.

## Materials and Methods

### Ethics statement

This study was carried out in strict accordance with the recommendations in the Guide for the Care and Use of Laboratory Animals of the National Institutes of Health. All animal procedures were performed according to a protocol approved by the Institutional Animal Care and Use Committee of the University of Pittsburgh (protocol number: 1002392). Appropriate sedatives, anesthetics and analgesics were used during handling and surgical manipulations to ensure minimal pain, suffering and distress to animals.

### Animals, treatments and sample collection

Nine Indian-origin rhesus macaques (*Macaca mulatta*) were used in the study. Four macaques were treated with the dual TLR7 and TLR9 antagonist DV056 (Dynavax Technologies), by weekly subcutaneous injections at 2 mg/kg, with 4 doses prior to and 8 doses after SIV infection. All macaques were infected by intravenous inoculation with 300 TCID_50_ SIVmac251 (provided by Preston Marx, Tulane National Primate Research Center). Bromodeoxyuridine (BrdU; Sigma) was administered by intravenous injection at 30 mg/kg at 24-h intervals for 3 days prior to collection of peripheral lymph nodes at day −7, 14 and 56 post infection, which were processed as previously described [23]. The quantitative real-time RT-PCR assay to determine SIV viral load was performed as previously described [25].

### Flow cytometry

The following monoclonal antibodies were used to label PBMC and lymph node cell suspensions and were purchased from BD Biosciences unless otherwise noted: CD3 (clone SP34-2), CD20 (eBiosciences, 2H7), CD14 (MøP9), CD123 (7G3), CD11c (S-HCL-3), HLA-DR (L243 or G46-6), CD163 (GHI/61), CCR7 (150503, R&D Systems), CD4 (L200), CD8 (RPA-T8), CD28 (CD28.2), CD38 (AT-1, Stem cell Technologies), CD95 (DX2), IRF-7 (H-246, Santa Cruz Biotechnology) and Ki67 (B56). Flow cytometric analysis and determination of blood pDC and CD4+ T cell counts were done as described [25], [41]. For detection of intracellular cytokines, cells were first labeled with surface-binding antibody and then fixed and permeabilized prior to incubation with antibody to TNF-α (MAb11) and IFN-α (225.C). In vivo incorporation of BrdU was detected using a BrdU-FITC staining kit (BD Biosciences). Dead cells were excluded using a Live/Dead viability kit (Invitrogen). Cells were run on a BD LSR-II flow cytometer system, collected with BD FACS Diva 6.0 software, and analyzed with FlowJo 8.8.7 (TreeStar). In general 1 million live cells were analyzed resulting in between 600 and 2,000 events of the respective mononuclear phagocyte subset after gating.

### Cell stimulations and pDC depletion

PBMC and lymph node cell suspensions were stimulated at 2.5×10^6^ cells/well for 7 h with iSIV at a capsid concentration of 200 ng/ml, virus-free microvesicles at 200 ng/ml, live SIVmac239 at a capsid concentration of 400 ng/ml (SIV preparations provided by Jeffrey D. Lifson, AIDS and Cancer Virus Program, SAIC-Frederick), live H7N3 influenza virus (provided by Ted M. Ross, University of Pittsburgh) at a multiplicity of infection of 5, or CpG-C oligodeoxyribonucleotide C274 (Integrated DNA Technologies) at a concentration of 5 ug/ml with and without prior incubation of cells with 1 µM DV056 for 1 h. Data generated using microvesicles as controls showed background cytokine production similar to unstimulated cells stained with isotype control antibody. In some experiments pDC were depleted from PBMC by labeling cells with PE-conjugated antibody to CD123 followed by anti-PE microbeads (Miltenyi) and then passing cells through a magnetic column (Miltenyi). For flow cytometric analysis, cytokine secretion was blocked by addition of 5 µg/ml brefeldin-A (BD Biosciences) after 2 h.

### Determination of secreted IFN-α

IFN-α in plasma and culture supernatants was measured using a commercial ELISA (multi-subtype IFN-α ELISA kit, PBL Biomedical) according to the manufacturer's instructions.

### Immunofluorescence of lymph node sections

Lymph nodes were prepared as described [23], cut to 7 µm –thick sections and stained overnight with mAb to IFN-α (MMHA2, PBL Interferon Source), TNF-α or isotype-matched antibody (BD Bioscience). Staining was developed using goat anti-mouse-HRP AlexaFluor 546 with tyramide signal amplification (Invitrogen). Sections were co-stained with rabbit antibody to CD3 (Dako) or biotinylated mouse antibody to CD123, CD163, CD1a (SK9, BioLegend), or isotype controls followed by donkey anti-rabbit Alexa488 secondary antibody or mouse HRP-streptavidin AlexaFluor 488. Nuclei were stained with Hoechst dye. Sections were visualized on an Olympus Fluoview 1000 confocal microscope and analyzed using Olympus FluoView Software. To quantify cytokine-expressing cells at least 10 non-overlapping regions were randomly imaged and cytokine-producing cells enumerated using image analysis software (Figure S1). Composite images were made by scanning entire lymph node sections using a Nikon 90i motorized epifluorescence microscope followed by digital reassembly using NIS-Elements.

### Gene expression analysis

Gene expression analysis was done as previously described [40]. Briefly, total RNA was extracted from previously cryopreserved PBMC and lymph node cells using an RNA micro kit (QIAGEN) and cDNA was generated with SuperScript First-Strand Synthesis System (Invitrogen). Quantification of ISG transcripts was performed by real-time RT-PCR in triplicate using TaqMan gene expression assays (Applied Biosystems). RCT threshold cycle (CT) values for each gene were normalized to the housekeeping gene ubiquitin using the formula gene expression = 1.8 (Avg CT Ubi – CT Gene)×100,000, where Ubi is the mean CT of triplicate housekeeping gene runs, Gene is the mean CT of duplicate runs of the gene of interest, and 100,000 is an arbitrary factor to raise values above 1. Primer sequences used were as follows: IRF7, CTGTTTCCGCGTGCCCT (forward), GCCACAGCCCAGGCCTT (reverse); Mx-b, GAGACATCGGACTGCAGAT (forward), GTGGTGGCAATGTCCACGTTA (reverse); 2.5 OAS, AGGGAGCATGAAAACACATTTCA (forward), TTGCTGGTAGTTTATGACTAATTCCAAG (reverse); GBP-1, TGGAACGTGTGAAAGCTGAGTCT (forward), CATCTGCTCATTCTTTCTTTGCA (reverse); and ISG-54, CTGGACTGGCAATAGCAAGCT (forward), AGAGGGTCAATGGCGTTCTG (reverse). Primers and probes for ISG20 (Hs00158122_m1) were provided by Applied Biosystems.

### Statistical analysis

Comparisons between treatment groups at a single time were performed by two- or three-way ANOVA. Profiles of measurements on animals assessed at multiple times were compared using mixed effects ANOVA, with a random effect for animal and fixed effects for time and other control variables. Where appropriate, tests were performed against baseline (Day 0 or −7) within treatment. For analysis of T cell subsets trend was tested by treating time as a discrete variable [71]. Tests of profiles across treatments were required to be significant before comparisons between treatments were made at and single time. All statistical analyses were performed using SAS v9.3 (SAS Institute).

## Supporting Information

Figure S1 **Quantification of cytokine-producing cells in lymph node sections.** Shown are representative images of IFN-α and TNF-α staining of untreated animals at day 14 and day 56 post infection, respectively. Portions of actual images are shown to highlight detail. To count nuclei, the fluorescence image of Hoescht-labeling in the original confocal image (left) are converted to 16 bit, gray scale images using the MetaMorph image analysis program (Molecular Devices). Parameters for nuclear width minimum and maximum and intensity of staining relative to background are then set using this program. An image is generated producing a gray scale dot over each nucleus (middle) and the corresponding nuclear count is determined. To count cytokine+ cells, the red/green/blue images of interest are opened in the ImageJ program (National Institutes of Health), and all images are adjusted for brightness and contrast. The cell counting application is launched and the image to be counted is initialized. Cytoplasmic staining that is associated with and surrounds at least 50% of a nucleus is identified and marked manually with a cursor creating a dot over the respective cell (right) and adding to a cumulative total of counted cells. The frequency of cytokine+ cells per 10,000 cells is then calculated using the formula: (Total number of cytokine+ cells ×10,000)/total number of nuclei.
(TIF)
