# Supplementary figures and images for: Blocking TLR7- and TLR9-mediated IFN-α Production by Plasmacytoid Dendritic Cells Does Not Diminish Immune Activation in Early SIV Infection
Source: PLoS Pathog. 2013 Jul 25;9(7):e1003530. doi: 10.1371/journal.ppat.1003530 (PMC3723633; doi:10.1371/journal.ppat.1003530)

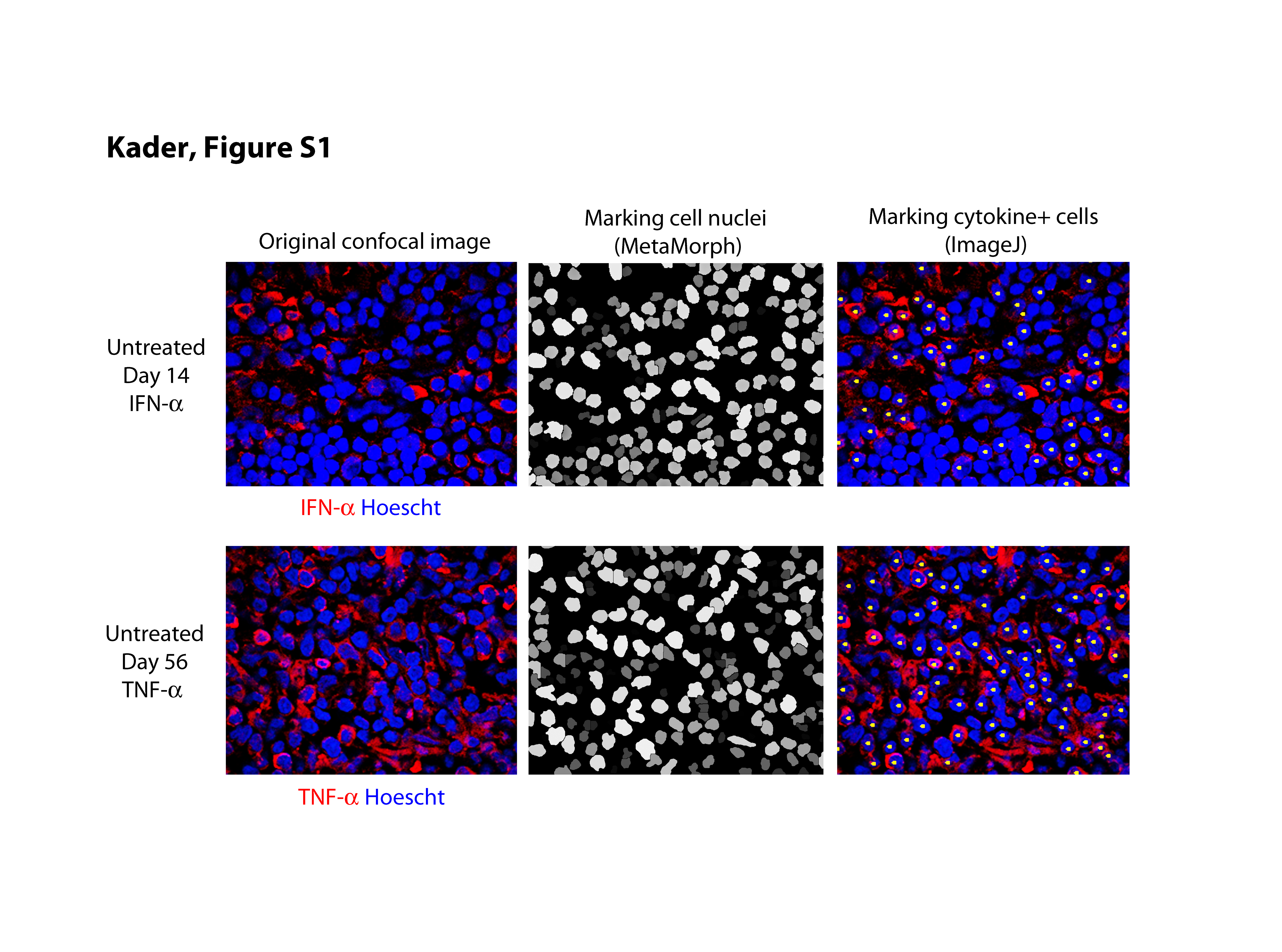

Supplement: Figure S1 — Quantification of cytokine-producing cells in lymph node sections. Shown are representative images of IFN-α and TNF-α staining of untreated animals at day 14 and day 56 post infection, respectively. Portions of actual images are shown to highlight detail. To count nuclei, the fluorescence image of Hoescht-labeling in the original confocal image (left) are converted to 16 bit, gray scale images using the MetaMorph image analysis program (Molecular Devices). Parameters for nuclear width minimum and maximum and intensity of staining relative to background are then set using this program. An image is generated producing a gray scale dot over each nucleus (middle) and the corresponding nuclear count is determined. To count cytokine+ cells, the red/green/blue images of interest are opened in the ImageJ program (National Institutes of Health), and all images are adjusted for brightness and contrast. The cell counting application is launched and the image to be counted is initialized. Cytoplasmic staining that is associated with and surrounds at least 50% of a nucleus is identified and marked manually with a cursor creating a dot over the respective cell (right) and adding to a cumulative total of counted cells. The frequency of cytokine+ cells per 10,000 cells is then calculated using the formula: (Total number of cytokine+ cells ×10,000)/total number of nuclei. (TIF) [file ppat.1003530.s001.tif]
